# Regional and organ-level responses to local lung irradiation in sheep

**DOI:** 10.1038/s41598-021-88863-8

**Published:** 2021-05-05

**Authors:** David Collie, Steven H. Wright, Jorge del-Pozo, Elaine Kay, Tobias Schwarz, Magdalena Parys, Jessica Lawrence

**Affiliations:** 1 The Roslin Institute and Royal (Dick) School of Veterinary Studies, University of Edinburgh, Midlothian, Edinburgh, EH25 9RG UK; 2 Present Address: Small Animal Clinical Sciences, School of Veterinary Medicine, University of Glasgow, Glasgow, UK; 3 Present Address: Department of Veterinary Clinical Sciences, University of Minnesota, St Paul, MN USA; 4 Present Address: Masonic Cancer Center, University of Minnesota, Minneapolis, MN USA

**Keywords:** Adverse effects, Radiotherapy, Cancer, Experimental models of disease, Translational research

## Abstract

Lung is a dose-limiting organ in radiotherapy. This may limit tumour control when effort is made in planning to limit the likelihood of radiation-induced lung injury (RILI). Understanding the factors that dictate susceptibility to radiation-induced pulmonary fibrosis will aid in the prevention and management of RILI, and may lead to more effective personalized radiotherapy treatment. As the interaction of regional and organ-level responses may shape the chronic consequences of RILI, we sought to characterise both aspects of the response in an ovine model. A defined volume of left pulmonary parenchyma was prescribed 5 fractions of 6 Gy within 14 days while the contralateral lung dose was constrained. Radiographic changes via computed tomography (CT) were documented to define differences in radio-exposed lung relative to non-exposed lung at d21, d63 and d171 (n = 2), and at d21, d147 and d227 (n = 2). Gross and histologic lung changes were evaluated in samples derived at necropsy examination to define the chronic pulmonary response to radiation. Irradiated lung demonstrated reduced radio-density and increased homogeneity as evidenced from texture based radiomic feature analysis, relative to the control lung. At necropsy, the radiation field was readily defined by pallor on the pleural surface, which was also evident on the cut surface of fixed lung specimens. The degree and homogeneity of pallor reflected the sparse presence of erythrocytes in alveolar septal capillaries of radiation-exposed lung. These changes contrasted with dilated and congested microvasculature in the contralateral control lung. Referencing data to measurements made in control lung volumes of sheep experiencing acute RILI indicated that interstitial collagen continues to deposit in the radio-exposed lung field. Overall lung vascularity increased during the chronic response, as evidenced by increased expression of endothelial cell marker (CD31); however, vascularity was consistently decreased in irradiated lung and was negatively correlated with lung collagen. Other organ-level responses included increased expression of alpha smooth muscle actin (ASMA), increased numbers of proliferating cells (Ki67 positive), and cells expressing the dendritic cell-lysosomal associated membrane protein (DC-LAMP) antigen. The chronic response to RILI in this model is effected at both the whole organ and local lung level. Whilst the long-term consequences of exposure to radiation involved the continued deposition of collagen in the radiation field, organ-level responses also included increased vascularization and increased expression of ASMA, Ki67 and DC-LAMP. Interrupting the interplay between these aspects may influence susceptibility to pulmonary fibrosis after radiotherapy. We advocate for the importance of large animal model systems in pursuing these opportunities to target local, organ-level and systemic mechanisms in parallel within the same subject over time.

## Introduction

Any patient receiving radiation therapy to the thorax is at risk for lung injury, affecting patients with a wide range of tumours, including lung cancer, breast cancer, oesophageal cancer, and some hematologic malignancies^1–4^. Up to one third of patients receiving radiotherapy will develop acute radiation pneumonitis (RP) within weeks to months of the last treatment^5–11^, and the majority of RP patients subsequently develop lung fibrosis months to years following treatment^1^. These acute and chronic toxicities, manifestations of radiation-induced lung injury (RILI), are a dose-limiting consideration in radiotherapy planning^2,12,13^. Patients who develop RP are at particular risk of radiation-induced lung fibrosis, which can have significant impacts on quality of life^14^, and for which there is no effective treatment^15^. Improved radiation technology carries the expectation that patient outcomes will also improve. It is therefore imperative that continued emphasis is placed on recognizing and mitigating chronic radiation-induced toxicities such as RILI, which are more likely to develop with longer survival. Understanding the transition between the acute and chronic phases of RILI may help define patient susceptibility to serious complications and offer opportunities for strategic prevention or therapeutic intervention.

Morgan & Breit (1995) distinguished classical from sporadic RP^16^. The former refers to dose-related lung toxicity characterised by local inflammatory responses that eventually lead to irreversible chronic lung fibrosis in the irradiated lung. In contrast, sporadic RP is unpredictable in terms of incidence and severity, with lung tissue beyond the radiation field commonly involved. Indeed, in their series of patients who received unilateral postoperative radiotherapy for breast cancer, 13 (75%) of the 17 patients had bilateral lymphocytosis on bronchoalveolar lavage (BAL).

Whilst RP generally resolves favourably, in 1–2% of patients the response can be life-threatening^6,17^. Further, in fatal RP, imaging can reflect RILI extending beyond the high dose area to involve the contralateral lung^18^, findings that have been confirmed across radiotherapy treatment modalities^19–23^. Further consideration indicates that RILI encompasses chronic toxicities that are also reflected beyond the radiation field. Venema et al., in a retrospective analysis of [18]F-fluoroestradiol (FES)-PET/CT scans, observed increased uptake consistent with pulmonary fibrosis in patients that had received thoracic irradiation an average of 7.3 years previously. In 23 of 48 patients, enhanced FES uptake was seen bilaterally, which was confirmed to extend beyond the boundaries of the radiation field^24^.

Such evidence for organ-level responses to local lung irradiation is also reflected in preclinical models. Ghobadi et al. demonstrated prominent perivascular oedema and a loss of endothelial cells in large and small vessels two weeks after proton irradiation targeting variable regions of the rat thorax^25^. Importantly, the pathology was also found in the shielded lung, indicating an organ-level vascular response. Due to the unique physical characteristics of protons, one of its primary reported advantages is that it reduces radiation dose to non-target tissues and may provide a therapeutic benefit to early stage lung cancer patients by reducing RILI^26,27^. This highlights that shielding tissues may not be sufficient to avoid injury. Indeed, out of field vascular remodelling has been reported to be virtually as severe as the in-field effects both early (2 weeks) and late (26 weeks) after irradiation^25,28^. Additional studies support these out-of-field effects. In a mouse model consisting of 20 Gy prescribed in a single dose to the upper right lung, significant neutrophil and macrophage infiltration was noted at 72 h with collagen accumulation at 26 weeks in the tissue outside the target tissue^29^. Lastly, unilateral lung irradiation in sheep was shown to impair the function of alveolar macrophages in both lungs one month after exposure^30^.

Evidence therefore indicates that the lung responds at the organ level to localised radiation injury. Morgan & Breit (1995) described the sporadic form as “entirely different” from the classical form^16^. The distinction, whilst undoubtedly relevant from a clinical perspective, risks ignoring possibly linked mechanisms that provide invaluable insight to the pathogenesis of RILI. Indeed, whilst much effort has traditionally focussed on the local events, whether inflammatory or fibrotic, little mechanistic insight has emerged in relation to organ-wide responses and how they relate to the former. Organ responses are potentially key to all aspects of the radiation response. For instance, the knowledge that in rats, local radiation influences endothelia throughout the lung^25,28^ has relevance to studies of interstitial lung diseases that demonstrate vascular remodelling even in areas unaffected by pathology^31–34^.

Further preclinical probing of these mechanisms would benefit from the facility to derive longitudinal samples from the same animal over a time span that encompasses the spectrum of RILI. Such a strategy reduces the inherent variability that besets the study of biology amongst individuals and it provides key data regarding individual responses at the local level, whether inside or outside the radiation field. The sheep as a large animal model provides this facility and over the course of many decades has helped to leverage insight at multiple levels, from physiological mechanisms to the pathophysiology of lung disease, including RILI^35–41^. Sheep are also amenable to the same imaging techniques, and interventional therapeutic modalities that are applicable in human medicine, and can be used to follow disease progression on time scales relevant to the human context.

We recently described events associated with the acute lung response to radiation in a sheep model, highlighting the mitigating influence of pre-radiotherapy administration of synthetic lamellar bodies^42^. The model demonstrated early deposition of collagen in the radiation field, a finding consistent with changes following radiation exposure in humans^43–45^. We sought to explore the hypothesis that chronic consequences of lung irradiation persist at the local level, and are also associated with organ-level responses. With aforementioned preclinical and clinical data indicating out of field fibrotic and vascular responses to local lung irradiation, we focussed our analysis on these aspects.

## Materials and methods

### Ethics statement

All experimental protocols were reviewed and approved by the local Roslin Institute ethical review process (The Roslin Institute Animal Welfare and Ethical Review Body) and all experimental procedures were performed in accordance with the provisions of the Animals (Scientific Procedures) Act 1986. During the course of the experimental protocols, all animals were assessed daily for evidence of adverse clinical effects relating to the procedures involved. Sample size (n = 4) for this pilot study was determined a priori using pragmatic estimation and was influenced by our previous observations regarding acute RILI^42^, suggesting that resolution of injury would progress from a consistent baseline un-associated with clinically evident adverse effect. Power calculations were not used to determine sample size. Inclusion criteria related to the absence of lung abnormality on thoracic computed tomography (CT) images, absence of grossly evident abnormality on baseline bronchoscopic examination, and baseline bronchoalveolar cytology values within normal ranges. The study was designed with due reference to the ARRIVE guidelines^47^.

### Animals

Four commercially sourced adult female Shetland sheep (bodyweight: 47.5 kg [46.0–49.0] median [range]) were included in this study. Identification of animals was by means of ear tags. Animals were housed for the duration of the study and otherwise maintained according to normal standards of farm animal husbandry. The sheep were treated with an anthelminthic before the study began.

### Experimental design

The experimental protocol relating to the preliminary assessment, and radiotherapy planning and treatment was identical to that used in our previous study^42^ in order to facilitate comparison with these earlier datasets. Briefly, in order to confirm the absence of pre-existing pulmonary disease, preliminary baseline bronchoscopic examination was conducted under inhalation anaesthesia to evaluate airway appearance, and derive bronchoalveolar lavage fluid samples for differential cytology values. Bronchial brush biopsy samples were also derived to assess for the expression of inflammatory cytokines (TGF beta, IL8 and IL1 beta).

At least four weeks after completing baseline assessment the sheep were re-anaesthetised and positioned in sternal recumbency in order to facilitate the acquisition of thoracic CTimages for subsequent radiation treatment planning. Radiation was prescribed to a defined volume of the left lung and administered as described below. Nebulised saline aerosol was also administered prior to each radiation treatment as described previously.

At t0 + 11d the sheep were re-anaesthetised and subjected to bronchial brush biopsy in the same manner as during the preliminary baseline evaluation. At t0 + 21d the sheep were again re-anaesthetised for the collection of bronchial brush biopsy samples, and for thoracic CT imaging.

Further CT imaging was conducted at t0 + 147d and t0 + 227d for two sheep (ED980 & ED982), and at t0 + 63d and t0 + 171d for the other two sheep (ED983 & ED984). On the day following the last CT, the sheep were euthanized by overdose of anaesthetic and presented for necropsy examination.

### Anaesthesia

Induction of anaesthesia was achieved using an intravenous injection of 6–8 mg/kg propofol (Fresenius propofol, 1%, Fresenius Kabi Ltd) and thereafter sheep were maintained under isoflurane anaesthesia using positive pressure ventilation (Model 708; Harvard Apparatus, Millis, MA).

### Image acquisition and three-dimensional conformal radiation treatment

Radiotherapy planning was undertaken following acquisition of a thoracic CT scan with the anaesthetised sheep positioned in sternal recumbency in a vacuum-formable mattress. Images were obtained with breath-hold to avoid motion artefact. Radiation treatment planning software (Eclipse v11.0, Varian Medical Systems Inc, Palo Alto, CA) was used for segmentation of organs at risk (OAR) and target volumes and for the creation of unique plans for each animal. All plans were created and approved by a board-certified veterinary radiation oncologist (JL). OARs included left lung, right lung, left mainstem bronchus, right mainstem bronchus, heart, oesophagus, trachea, spinal cord, reticulum, rumen, abomasum, and omasum. A clinical target volume (CTV) was manually created in the left caudal lung lobe with the cranial aspect located 6 cm caudal to the tracheal bifurcation and extending to within 1 cm of midline and within 0.5 cm away from the body wall. A planning target volume (PTV) was created by expanding the CTV by an isotropic 3 mm expansion but cropped to ensure it confined to the left lung. The PTV was retracted from midline at least 0.5 cm in order to limit scatter dose to the right lung and the overall PTV volume was constrained to < 15% of the total lung volume (mean 10.7%, range 9.1–11.8%). All sheep were treated using a linear accelerator (Varian Clinac 2100 C/D, Varian Medical Systems) and 6MV photons at a dose rate of 600MU/minute. Two non-coplanar beams were used and 5 fractions of 6 Gy were prescribed to 100% of the dose at isocentre within the PTV. Fractions were administered at 3- or 4-day intervals (Table 1) using the same breath hold technique that was used for CT simulation. Orthogonal electronic portal images were taken immediately prior to irradiation and used to adjust the sheep’s position and document accurate positioning for photon irradiation. Planning goals included that at least 95% of the PTV was covered by 27 Gy, the mean PTV dose was greater than 29 Gy, and the maximum dose was 33 Gy. Due to heterogeneity of dose deposition within aerated lung, dose to the right lung could not be entirely avoided. To minimize the impact of scatter radiation to the right lung, the mean dose to right lung was required to be less than 1 Gy, with dose greater than 0.5 Gy limited to the midline portion of the caudal right parenchyma, medial to the planned region for bronchial brush and tissue sample collection. Secondary planning goals aimed to limit the maximum dose to the right mainstem bronchus to 1 Gy and to constrain the volume of right lung receiving 1 Gy or higher to less than 10% (V1 < 10). Dose constraints to abdominal structures were identical to those previously described^42^.

**Table 1:** Radiotherapy planned dose parameters for the clinical target volume (CTV), planning target volume (PTV), right lung (RL), left lung (LL), and right mainstem bronchus (RMB) of sheep prescribed 30 Gy irradiation with 6MV photons to the PTV within the caudal LL.

Parameter	ED980	ED982	ED983	ED984
Duration of treatment (days)	14	14	14	14
CTV (cm^3^)	81.5	114.3	83.3	88.1
**Dose to CTV (Gy)**				
Min	25.98	26.24	26.41	26.72
Mean	29.89	29.95	29.93	29.74
Max	32.64	31.68	31.47	31.57
PTV (cm^3^)	134.9	174.5	133.4	145.7
**Dose to PTV (Gy)**				
Min	25.25	25.41	25.37	25.21
Mean	29.76	29.85	29.81	29.46
Max	32.99	31.82	31.74	31.79
LL Volume (cm^3^)	545.0	666.7	502.4	709.7
**Dose to LL (Gy)**				
Min	0.25	0.24	0.22	0.10
Mean	20.34	19.60	20.46	17.48
Max	33.85	32.67	32.34	32.50
RL Volume (cm^3^)	665.3	941.5	629.8	888.3
**Dose to RL (Gy)**				
Min	0.14	0.11	0.13	0.09
Mean	0.48	0.62	0.54	0.52
Max	1.61	11.1	4.32	8.87
RL Volume > 1 Gy (V1; %)	3.0	11.7	7.9	7.0
**Dose to RMB (Gy)**				
Min	0.4	0.6	0.5	0.4
Mean	0.7	0.9	0.7	0.7
Max	1.0	1.3	0.9	1.0
**Dose to LL (Gy)**				
Min	0.25	0.24	0.22	0.10
Mean	20.34	19.60	20.46	17.48
Max	33.85	32.67	32.34	32.50

### CT Image analysis

For each sheep the CT image slice corresponding to the isocentre of the radiation field was identified and a 10 pixel deep subpleural region, extending around the periphery of the lung field selected using the brush selection tool in ImageJ^46^. The following 1st order statistics were calculated for each region of interest (Area (mm^2^), the mean grey value (Hounsfield units, HU), HU SD, HU CV, HU min, HU max, Skewness and kurtosis). The process was then applied to the right lung field in the same image slice. Texture analysis of CT images involved manual segmentation of the lungs using ImageJ^46^. A threshold was then applied to select for image pixels representing pulmonary parenchyma in the range − 1024 to − 242. The segmented and thresholded image of each lung was converted to a mask. Subtraction of the mask from the original image enabled selection of regions representing pulmonary parenchyma with background represented by zero values. These images were saved as text images in order to facilitate conversion of background values to NaN (Not a Number) using Microsoft Excel (2016), and the resultant matrix imported as a text image to ImageJ before being saved as a tagged image format (TIFF) file. Each image was opened in Matlab (R2019b) and the graycomatrix function from the image processing toolbox employed to create grey-level co-occurrence matrices (GLCM) specifying the frequencies of combinations of image intensities. Grey scale intensities were scaled to integer values between 1 and 64, offset was set to one pixel, and each GLCM was created symmetric across its diagonal. Pixel combinations directed at angles of 0, 45, 90 and 135 degrees were evaluated. The graycoprops function was used to normalize each GLCM and calculate contrast, correlation, energy and homogeneity statistics, with data presented as the average of the four directions. A fifth measure of image entropy was calculated using the histogram function in ImageJ, and computing entropy as -sum(pi * log_2_(pi)) where p is the probability of an image grey level i in the histogram.

### Bronchoalveolar lavage

Bronchoalveolar lavage fluid (BALF) was collected following wedge positioning of the bronchoscope (Model FG-15 W; Pentax UK Ltd.) in the segmental bronchus of the right apical lobe, and sequential instillation and recovery of 20 ml aliquots of phosphate buffered saline (PBS). BALF samples were kept on ice until centrifugation at 400 g for seven minutes to separate out the cellular fraction. The pellet was re-suspended PBS and the total cell number counted using a Neubauer haemocytometer. Cyto-centrifuge slides were prepared and stained using Leishman stain for differential counts on 500 cells.

### Bronchial brush biopsy

Disposable cytology brushes (Type 152R, Conmed UK Ltd) were used to collect bronchial epithelial cell samples from selected segmental bronchi. Cells were collected by agitating the brush through a modified pipette tip into cold sterile phosphate buffered saline (PBS, Sigma D8537), followed by centrifugation at 10,000×g for 5 min.

### Quantitative Real Time PCR

Cell pellets were re-suspended in RLT buffer (Qiagen 74106) containing 1% β mercaptoethanol and stored at − 80 °C until extraction. All samples were run through Qiashredder columns (Qiagen 79656) and RNA extractions were performed using RNeasy mini kit (Qiagen 74106) with DNase treatment using RNase free DNase set (Qiagen 79254). RNA was quantified and quality assessed on Nanodrop. cDNA was prepared from 400 ng RNA using Transcriptor First Strand cDNA Synthesis kit (Roche 04 896 866 001) and random hexamer primers. Quantitative Real Time PCR was performed using the Lightcycler 480 (Roche UK) with 2.5 µl cDNA in LightCycler 480 Sybr Green I Master (Roche 04 887 352 001) and specific primers (Table 2). Advanced relative quantification was calculated using Lightcycler 480 SW1.5 programme. Standard curves for each gene were generated from pooled ovine alveolar macrophage cDNA. Melt curve analysis showed a single peak for all samples. PCR efficiency was in the range of 1.95 to 1.99.

**Table 2:** qPCR conditions and primer sets.

Gene	Pre incubation	Amplification	Melting curve
Ovine ATPase and IL1 beta	95 °C for 15 min	50 cycles of 94 °C 15 s 52 °C 30 s 72 °C 30 s 80 °C 5 s	95 °C 5 s ramp 4.4 40 °C 1 min ramp 2.2 97 °C continuous 0.11 72 °C 10 min ramp 2.2
Ovine TGF beta	50 °C 2 min 95 °C 8.5 min	40 cycles of 95 °C 15 s 60 °C 1 min	95 °C 1 min ramp 4.4 55 °C 1 min ramp 2.2 97 °C continuous 0.11
Ovine IL8	45 °C 30 min 95 °C 10 min	40 cycles of 94 °C 20 s 55 °C 30 s 72 °C 30 s	95 °C 5 s ramp 4.4 55 °C 1 min 2.2 95 °C continuous 0.29

	Forward 5′–3′	Reverse 5′–3′	
Ovine ATPase Ovine	GCTGACTTGGTCATCTGC	CAGGTAGGTTTGAGGGGATAC	
TGF beta	CTGAGCCAGAGGCGGACTAC	TGCCGTATTCCACCATTAGCA	
Ovine IL1 beta	CCCATTAATGAAGTGATGGC	CTAGGGAGAGAGGGTTTCCA	
Ovine IL8	CACTGCGAAAATTCAGAAATCATTGTTA	CTTCAAAAATGCCTGCACAACCTTC	

### Necropsy

Following euthanasia by intravenous injection of barbiturate, and removal of the heart and lungs, the pulmonary circulation was perfused with PBS and the lungs photographed.

### Inflation fixation

The lungs were fixed by airway instillation of 10% neutral buffered formalin at a constant pressure of 3.0 kPa for 7 days.

### Gross tissue sampling

After fixation each lung was transversely sliced into 1 cm thick tissue slices, and the slices arranged in consecutive order for photographing. A tissue block was dissected from each contiguous slice and a further photographic image of the slices captured with their selected blocks still in situ to record spatial origin of each block and facilitate registration with respect to the radiation field on the axial CT images. Tissue blocks were submitted for standard histological processing and paraffin embedding.

### Block selection

The formalin-fixed paraffin embedded (FFPE) tissue block incorporating the isocentre of the planning target volume was selected (LL), as was the corresponding block from the right contralateral control lung (RL).

### Histochemical and immunohistochemical staining

Sections cut from the above blocks were stained with haematoxylin–eosin (H&E) and the collagen stain, Picrosirius Red, as well as being immunostained with antibodies specific for alpha-smooth muscle actin (ASMA), dendritic cell-lysosomal associated membrane protein (DC-LAMP), CD31 (PECAM-1; endothelial cell marker) and Ki67. Immunohistochemical methods for ASMA, DC-LAMP and Ki67 were as previously described^42^. ASMA is expressed by myofibroblasts, cells implicated as the source of extracellular matrix in lung fibrosis, and DC-LAMP is expressed by type II pneumocytes (AEC2), the main source of vascular endothelial growth factor (VEGF) in the lung parenchyma. Cell proliferation is a feature of the tissue response to injury, and Ki67 was used to quantify this aspect of repair.

CD31—antigen retrieval was achieved using pre-warmed 0.05% Pepsin (Sigma P7000) in 10 mM HCl, digestion at 37 °C for 1 h. Non‐specific binding was blocked with 10% Normal Donkey Serum (Sigma D9663) in 1% Bovine Serum Albumin (Sigma A4503)/PBS + 0.05% Tween 20 (10%NDS/BSA/PBS-T). Primary antibodies CD31 (PECAM-1) clone CO.3E-1D4 (Novus Biologicals NB100-65900) and Normal Mouse IgG (Sigma M5284) were diluted to 10 µg/ml in 1:10 blocking solution (10% NDS/BSA/PBS-T diluted in BSA/PBS-T) and incubated overnight at 4 °C. Detection was achieved using biotinylated horse anti mouse (Vector BA2001) and Streptavidin peroxidase polymer (Sigma S‐2438) followed by DAB substrate (Vector SK‐4105) with Haematoxylin counterstain.

### Qualitative histopathological evaluation

All H&E stained sections were scanned on a whole slide scanner (Nanozoomer, Hamamatsu, Japan) to acquire whole slide images (WSI) at × 40 magnification. A board certified veterinary pathologist (JDP), blinded to the source of the slides, conducted a qualitative appraisal of the histopathology.

### Quantitative histological analyses

Within ImageJ^46^, the NDPITools custom extract to TIFF/mosaic plugin was used to extract each ndpi image file at × 40 resolution to multiple TIFF images. Every image field containing parenchyma (including airways no larger than respiratory bronchioles) was manually selected from these extracted files and then converted to OME-TIFF using an ImageJ recursiveTiffConvert macro to engage the Bio-Formats exporter function.

For immunohistochemical and histochemical stains, targets were evaluated as expression per unit tissue area. The parenchymal × 40 OME-TIFF files were batch processed using macros employing the colour devolution plugin for detecting the area of red-stained collagen in Picrosirius Red-stained sections, the area of DAB stain in ASMA, and CD31-immunostained sections, and the number of DAB-stained DC-LAMP positive cells.

### Statistical analyses

Data are expressed as mean ± standard deviation, and graphically presented in the form of box plots, scatterplots and histograms. The normality of distribution of continuous data was assessed using a Kolmogorov_Smirnoff test, and data transforms employed where appropriate.

In order to minimise the confounding influence of between-animal variation in measurements derived from CT images, absolute levels of variables were expressed in terms of the difference between the radio-exposed (Rx) and contralateral (CON) lung. These differences were used in evaluating time dependent change (baseline (d-21), acute (d21), and chronic (d171/d227)) using repeated measures ANOVA, with sheep ID included as a random factor.

Preliminary appraisal of quantitative indices of histochemistry and immunohistochemistry suggested the value of providing a second benchmark, in addition to the chronically responding contralateral lung, against which to compare the chronic changes wrought by radiation. We accessed archived material from our previous study in which the acute response to an identical radiation prescription was evaluated in six sheep^42^, and we employed the same measures to sections derived from the un-irradiated lung tissues, which were grossly and histologically normal. One way ANOVA was employed to determine whether the means of histologic indices differed between lung sections derived from chronic response radio-exposed (Rx) and contralateral (CON) lung, and equivalently handled archived material derived from the contralateral (CON) lung of sheep undergoing an acute radiation response.

The degree of relationship between measurement variables was assessed through calculation of the Pearson product moment correlation coefficient. Statistical analyses were completed using Minitab Version 17 statistical software (Minitab, State College, PA, USA).

## Results

Baseline bronchoalveolar lavage cytology was within normal ranges for all cell types (cells/ml (× 10^6^): 3.11 [2.24–3.64], percent neutrophils: 1.6 [0.6 – 6.0], percent macrophages: 89.7 [83.4–94.4], percent lymphocytes: 5.5 [4.6–8.4], percent mast cells: 0.5 [0.2–0.8], percent eosinophils: 0.5 [0–4], percent other: 0.5 [0–1]) (median [range]).

Radiation dose parameters are outlined in Table 1, demonstrating a mean dose to the PTV in all sheep greater than 29 Gy with limited scatter dose to the control (right) lung and mainstem bronchus. All sheep tolerated treatment well without radiation adverse events noted during the follow-up period. Average values for each of the 1st order statistics derived from radio-exposed (Rx) and contralateral (CON) lungs imaged at baseline (d-21), at d21, and just prior to necropsy (d171 or d227) are presented in Table 3. Repeated measures ANOVA indicated that there was a significant time dependent influence on measurements reflecting the difference between radio-exposed relative to contralateral control lung. These measurements comprised the mean and standard deviation of pixel grey level intensity (P < 0.000 and P < 0.008 respectively) and reflected a relative increase in the radio-exposed lung at d21, followed by a decrease at 171–227 days after irradiation.

**Table 3:** 1st order statistics describing the distribution of individual pixel values in lung slice images derived from CT scan images of radio-exposed (Rx) and contralateral control (CON) lung at time points prior to (d-21), and following radiation exposure (d21, and d171/227).

Sheep	Lung	Timepoint (d)	Area (mm2)	HU mean	HU SD	HU min	HU max	Skewness	Kurtosis
ED980	CON	− 21	1833	− 490	131	− 952	229	0.68	1.63
		21	1906	− 499	183	− 980	207	0.59	− 0.05
		227	2102	− 516	231	− 1003	233	0.69	− 0.25
	Rx	− 21	1843	− 503	133	− 815	51	0.70	0.43
		21	1955	− 378	229	− 850	164	0.19	− 1.27
		227	1858	− 605	158	− 919	420	2.06	5.43
ED982	CON	− 21	2739	− 693	79	− 1023	− 132	1.57	6.45
		21	1583	− 436	136	− 828	205	0.66	0.73
		227	1579	− 465	255	− 1020	266	0.69	− 0.51
	Rx	− 21	2450	− 642	104	− 1024	189	1.36	4.95
		21	1496	− 239	164	− 871	174	− 0.49	− 0.38
		227	1775	− 579	128	− 894	642	1.51	6.41
ED983	CON	− 21	1765	− 564	109	− 935	27	0.52	0.84
		21	1946	− 652	88	− 932	18	0.54	1.90
		171	1492	− 475	114	− 849	28	0.31	0.31
	Rx	− 21	1752	− 588	106	− 916	105	0.85	2.48
		21	1873	− 529	144	− 961	88	0.28	− 0.04
		171	1441	− 634	132	− 956	225	1.26	3.96
ED984	CON	− 21	1747	− 713	96	− 1004	− 198	0.56	1.47
		21	1607	− 659	98	− 945	− 50	0.69	1.98
		171	1442	− 574	198	− 988	558	1.55	3.00
	Rx	− 21	1817	− 681	123	− 1007	− 25	0.69	0.67
		21	1872	− 531	155	− 909	165	0.57	0.25
		171	1698	− 687	109	− 1018	− 50	0.50	0.85

Texture analysis statistics relating to GLCMs derived from CT scan images of radio-exposed (Rx) and contralateral control (CON) lung at time points prior to (d-21), and following radiation exposure (d21, and d171/227) are presented in Table 4. Repeated measures ANOVA applied to the differences in GLCM statistics derived from radio-exposed relative to contralateral control lung indicated that there was a significant time dependent influence on homogeneity of the GLCM reflecting a relative reduction of the radio-exposed lung at d21, and a subsequent increase 171–227 days after irradiation.

**Table 4:** Texture analysis statistics relating to grey level co-occurrence matrices derived from CT scan images of radio-exposed (Rx) and contralateral control (CON) lung at time points prior to (d-21), and following radiation exposure (d21, and d171/227).

Sheep	Lung	Timepoint (d)	Homogeneity	contrast	energy	correlation	entropy
ED980	CON	− 21	0.219	102.7	0.0012	0.50	6.30
		21	0.236	83.0	0.0011	0.68	6.44
		227	0.217	109.0	0.0010	0.62	6.40
	Rx	− 21	0.216	108.0	0.0012	0.47	6.29
		21	0.222	101.6	0.0010	0.60	6.40
		227	0.261	74.1	0.0020	0.58	6.00
ED982	CON	− 21	0.256	88.0	0.0030	0.25	5.88
		21	0.256	68.9	0.0016	0.66	6.04
		227	0.224	105.8	0.0013	0.56	6.32
	Rx	− 21	0.251	94.1	0.0021	0.43	6.20
		21	0.247	93.4	0.0017	0.69	5.95
		227	0.239	82.8	0.0017	0.50	6.04
ED983	CON	− 21	0.250	83.3	0.0017	0.57	5.96
		21	0.236	82.7	0.0018	0.45	6.11
		171	0.243	73.0	0.0015	0.58	5.90
	Rx	− 21	0.258	77.0	0.0018	0.60	5.95
		21	0.232	89.2	0.0010	0.63	6.52
		171	0.253	80.6	0.0015	0.65	6.11
ED984	CON	− 21	0.246	82.1	0.0020	0.47	5.92
		21	0.227	98.4	0.0019	0.36	5.84
		171	0.242	85.0	0.0017	0.58	6.00
	Rx	− 21	0.245	82.5	0.0016	0.57	6.10
		21	0.220	101.9	0.0011	0.56	6.18
		171	0.254	79.0	0.0018	0.58	5.92

Levels of cytokine (TGF beta, IL8 and IL1 beta) gene expression were analysed by repeated measures ANOVA to determine the influence of time, with sheep identity included as a random factor. Gene expression did not differ significantly (P > 0.05) between time points (Table 5).

**Table 5:** Levels of expression of cytokines (TGF beta, IL8 and IL1 beta) in bronchial brushing samples.

	TGF beta	IL8	IL1 beta
Baseline	0.45 ± 0.66	0.60 ± 0.95	0.34 ± 0.61
d11	0.40 ± 0.49	0.25 ± 0.32	0.14 ± 0.20
d23	0.32 ± 0.32	1.26 ± 2.54	0.40 ± 0.80

Levels of gene expression (mean ± SD) reflect ratios relative to a housekeeping gene (ATPase). The data were analysed by repeated measures ANOVA to determine the influence of time, with sheep identity included as a random factor. Levels of cytokine expression did not differ significantly (P > 0.05) between time points.

**Figure 1:**
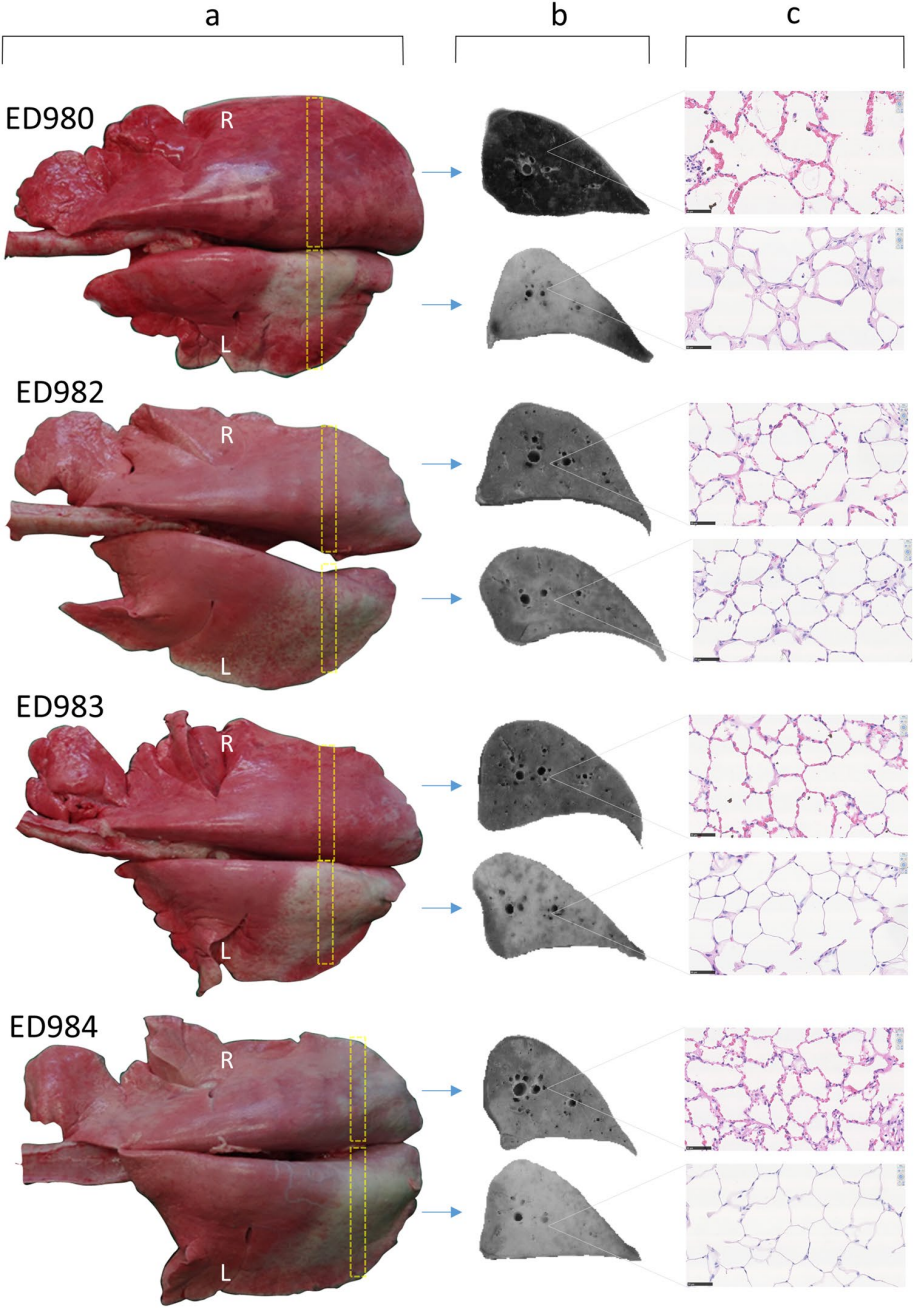
Gross and histological features associated with chronic RILI. Column (**a**): Gross features evident at post mortem examination demonstrated pallor on the pleural surface overlying the radio-exposed volume of the left (L) lung which was variable in extent, ranging from sparse mottling (ED982) through to a more uniform distribution (ED980, ED983 & ED984), with discernible thickening and evident contraction in one case (ED980). Column (**b**): Following inflation fixation each lung was sectioned into 1 cm thick transverse slices. A representative greyscale image of a slice (yellow boxes with dashed borders) derived from the centre of the radiation field, and the contralateral control lung (R) is depicted. The images demonstrate that the variable pallor evident on the pleural surface extends through the lung substance, being most noticeable and uniform in the lungs of ED980 and ED984, more mottled in ED983, and less evident in ED982. Column (c): Representative histological images captured from haematoxylin and eosin-stained sections demonstrate the association between pallor and the absence of erythrocytes in the peripheral microvasculature (scale bars = 50 μm).

**Figure 2:**
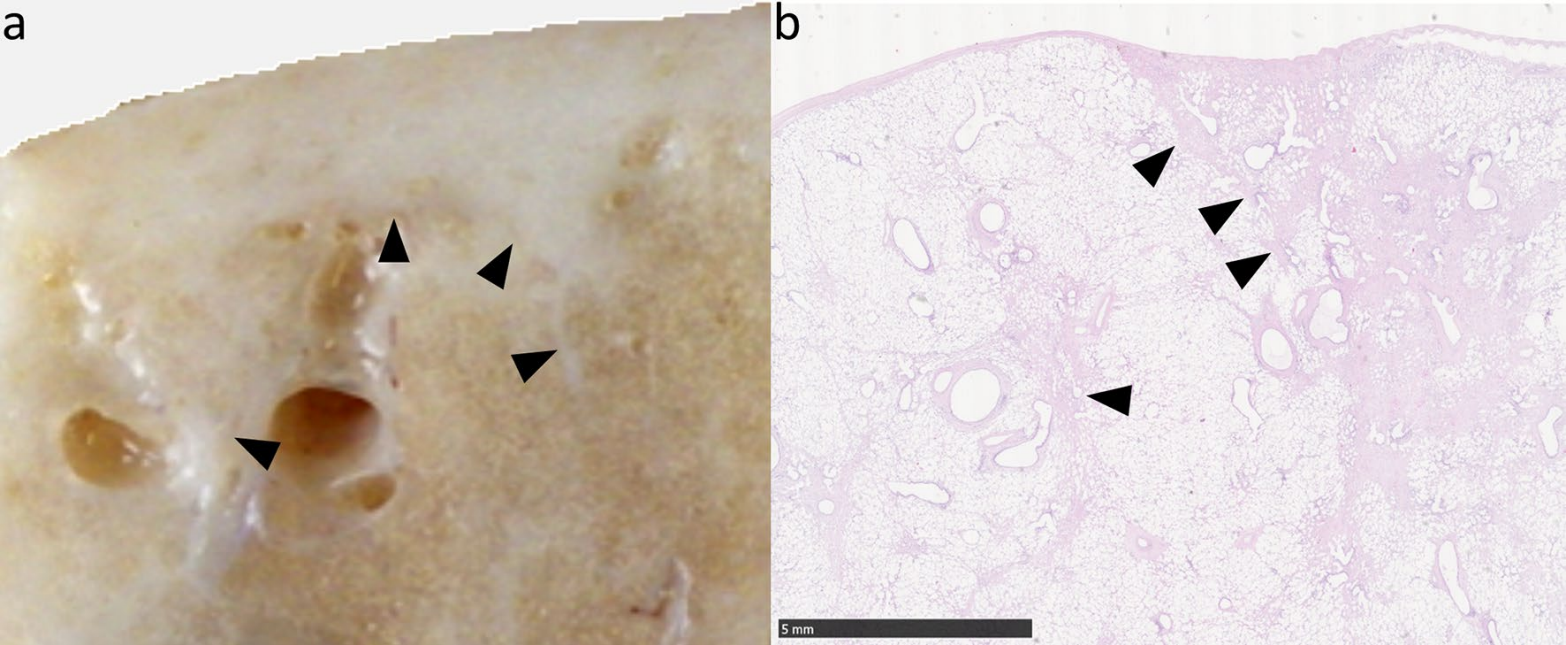
Gross and histological evidence of fibrotic change. (**a**) Photograph of a piece of fixed lung from ED980. The pleura, which is grossly thickened with fibrosis, lines the upper margin and fibrotic change also extends into the lung substance along septal boundaries (arrowheads). (**b**) Photomicrograph of a haematoxylin and eosin stained histological field from the same block. The nature of the grossly evident fibrosis is confirmed (arrowheads) (scale bar = 5 mm).

Gross features evident at post-mortem examination indicated a range of responses amongst the four sheep (Fig. 1a). Consistently the pleural surface overlying the radio-exposed lung volume exhibited pallor, which ranged from sparse mottling (ED982) through to a more uniform distribution (ED980, ED983 & ED984) with discernible thickening and evident contraction in one case (ED980). Following gross sectioning of the fixed lungs it was noted that the radio-exposed lung volumes exhibited pallor to a variable degree relative to un-irradiated areas of the same lung, and the contralateral control lung (Fig. 1b). This pallor was most noticeable and uniform in the lungs of ED980 and ED984, more mottled in ED983, and less evident in ED982. Closer examination of the cut lung surface at the pleural margins of ED980 revealed uniform white thickening indicative of organised fibrosis (Fig. 2).

Histopathological evaluation revealed the most marked changes were found in the radio-exposed lung sections from ED980 and ED983. These changes included focally extensive alveolar fibrosis with peripheral histiocytosis and mild multifocal alveolar oedema/fibrin. ED984 demonstrated very mild fibrotic change, and no evidence of fibrosis was found in lung sections from ED982. Inflammation was considered very subtle if present at all, both in terms of peribronchial, peribronchiolar and perivascular locations and in areas of alveolar fibrosis. Type II pneumocytes and airway epithelial cells were considered mostly normal. Several instances of parasitism were noted, generating background lesions in several of the sheep and pigment laden intra-alveolar histiocytes were noted in several sheep. Lung tissue sections from radio-exposed lung were characterised by a paucity of erythrocytes in the alveolar microvasculature, whereas the microvasculature of the contralateral lung was markedly congested (Fig. 1c).

**Figure 3:**
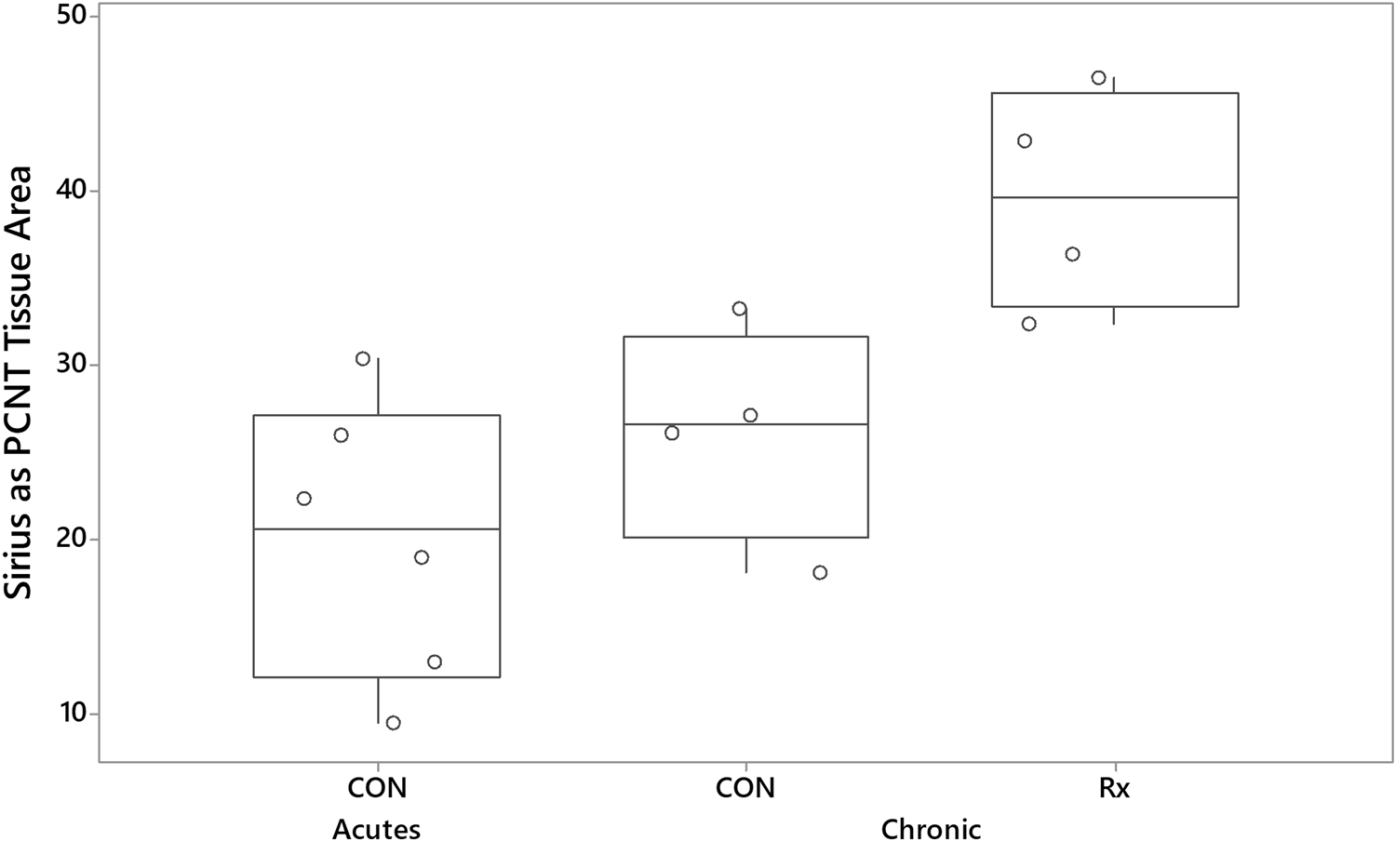
Boxplot illustrating Picrosirius Red stain expressed as a percentage of tissue area. Each symbol represents the average of measurements made in every available parenchymal field found in sections derived from the radio-exposed (Rx) and contralateral control (CON) lung of sheep experiencing chronic responses to radiation (Chronic), and contralateral control lungs of six sheep experiencing acute responses to an identical radiotherapy protocol (CON, Acutes; 23 days after the last radiation dose). The data illustrates an increase in collagen in the radio-exposed lung (Picrosirius Red stain). The top of each box represents the third quartile and the bottom the first quantile, the upper and lower whiskers extend to the highest and lowest data values within the upper and lower limits, respectively, and the central line represents the median.

An increase in the area of Picrosirius Red stain was a consistent feature of the chronic response to radiation (Fig. 3). One-way ANOVA indicated that Picrosirius Red stain expressed as a percentage of tissue area was significantly (P = 0.004) increased in the radio-exposed lungs of sheep responding chronically to radiation relative to levels found in contralateral control lungs of sheep responding acutely to radiation. The increase in Picrosirius Red stain in radio-exposed relative to contralateral lung was a consistent finding in the chronic response.

**Figure 4:**
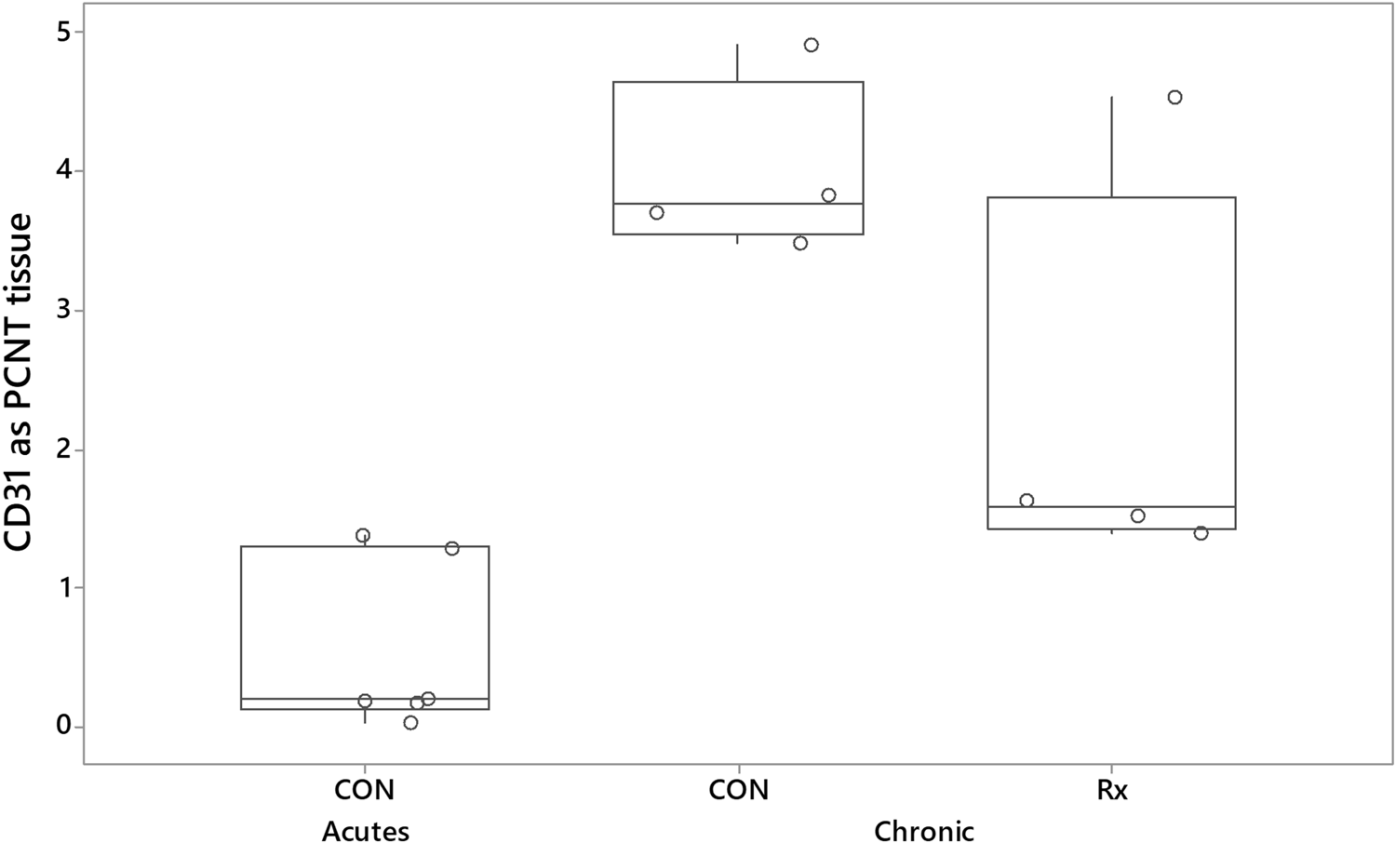
Boxplot illustrating CD31 stain expressed as a percentage of tissue area. The derivation of CD31 measurements was as described for Fig. 3. The data illustrates that, relative to levels seen in the control lungs of sheep undergoing an acute radiation response (CON, Acutes), there was increased expression of endothelial cell marker throughout the lungs of sheep undergoing a chronic radiation response. With the exception of one sheep (ED982), radiation exposure was associated with a decrease in expression of CD31 in the radio-exposed lung (Rx) relative to the contralateral control lung (CON).

**Figure 5:**
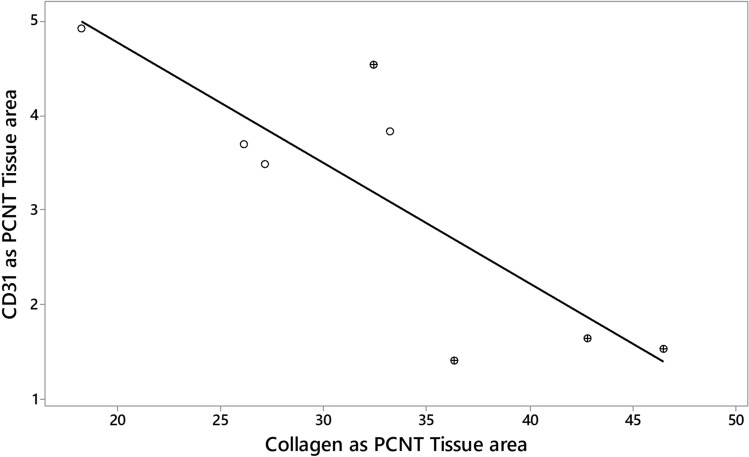
Relationship between collagen and CD31 expression in sheep during the chronic response to radiation. For each sheep the average values for collagen (Picrosirius Red) and CD31 stain area were calculated for each radio-exposed (⊗) and contralateral control (Ο) lung and plotted against each other. A significant negative association existed between these variables (Pearson correlation = − 0.829; P-Value = 0.011).

The expression of CD31 was significantly increased in both the chronically responding radio-exposed and contralateral control lungs relative to expression found in acutely responding control lungs from a separate cohort (Fig. 4). One-way ANOVA indicated that CD31 expression was significantly (P < 0.001) increased in both radio-exposed and contralateral control lungs of sheep responding chronically to radiation relative to levels found in control lungs of sheep responding acutely to radiation. A significant negative relationship existed between CD31 and collagen stain area (Pearson correlation = − 0.829; P = 0.011) (Fig. 5).

**Figure 6:**
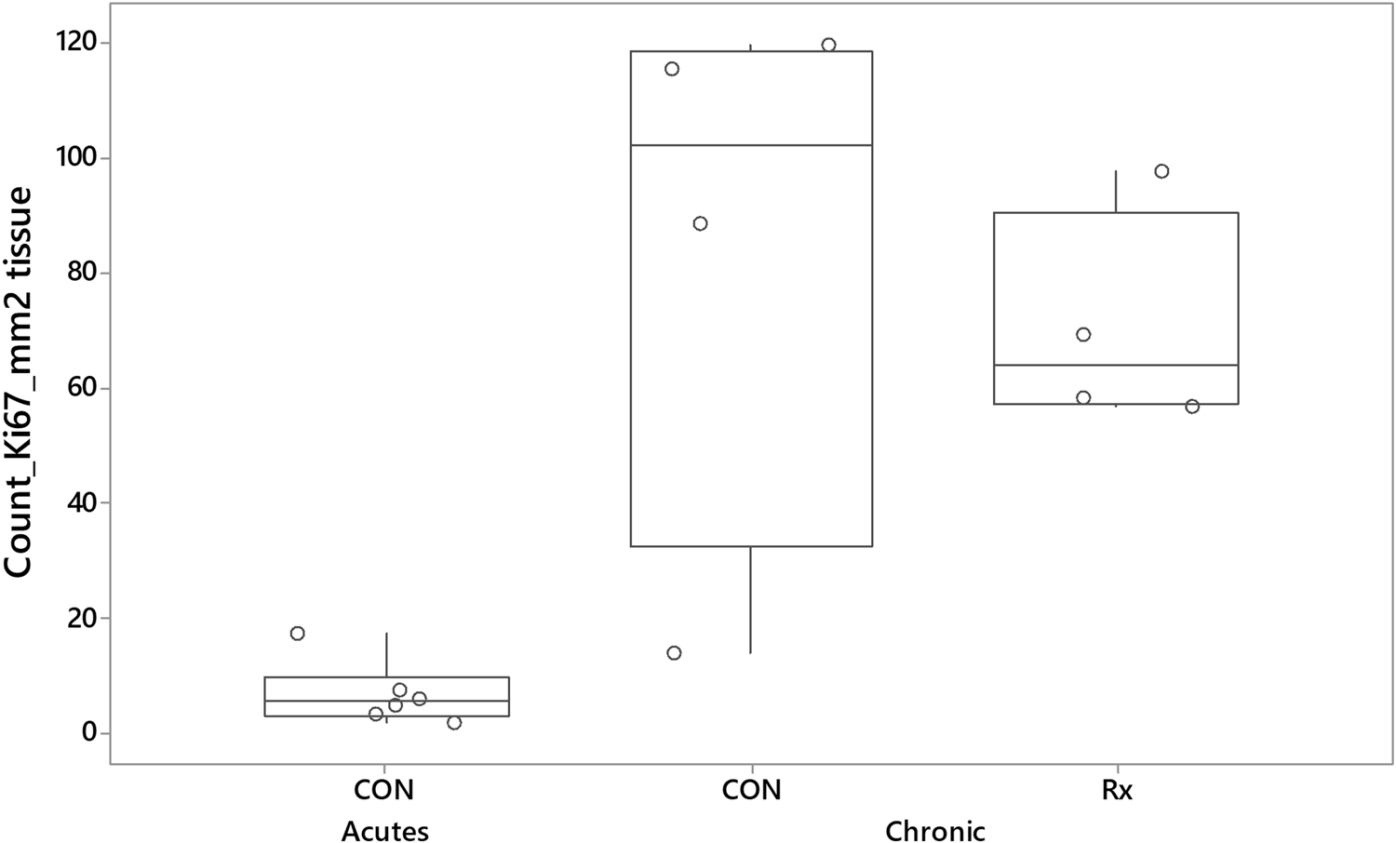
Boxplot illustrating the mean number of Ki67 positive cells expressed per mm^2^ lung tissue. The derivation of mean numbers of Ki67 positive cells was as described for Fig. 3. The data illustrates that, relative to levels seen in the control lungs of sheep undergoing an acute radiation response (CON, Acutes), there was increased numbers of Ki67 positive cells throughout the lungs of sheep undergoing a chronic radiation response.

One-way ANOVA indicated that proliferating cells were significantly (P = 0.001) increased in both radio-exposed and contralateral control lungs of sheep responding chronically to radiation relative to levels found in control lungs of sheep responding acutely to radiation (Fig. 6). Although not reaching significance (P = 0.078), a negative relationship (r^2^ = − 0.65) between Ki67 and Picrosirius Red was noted, suggesting that areas with increased collagen deposition featured less cell proliferation.

**Figure 7:**
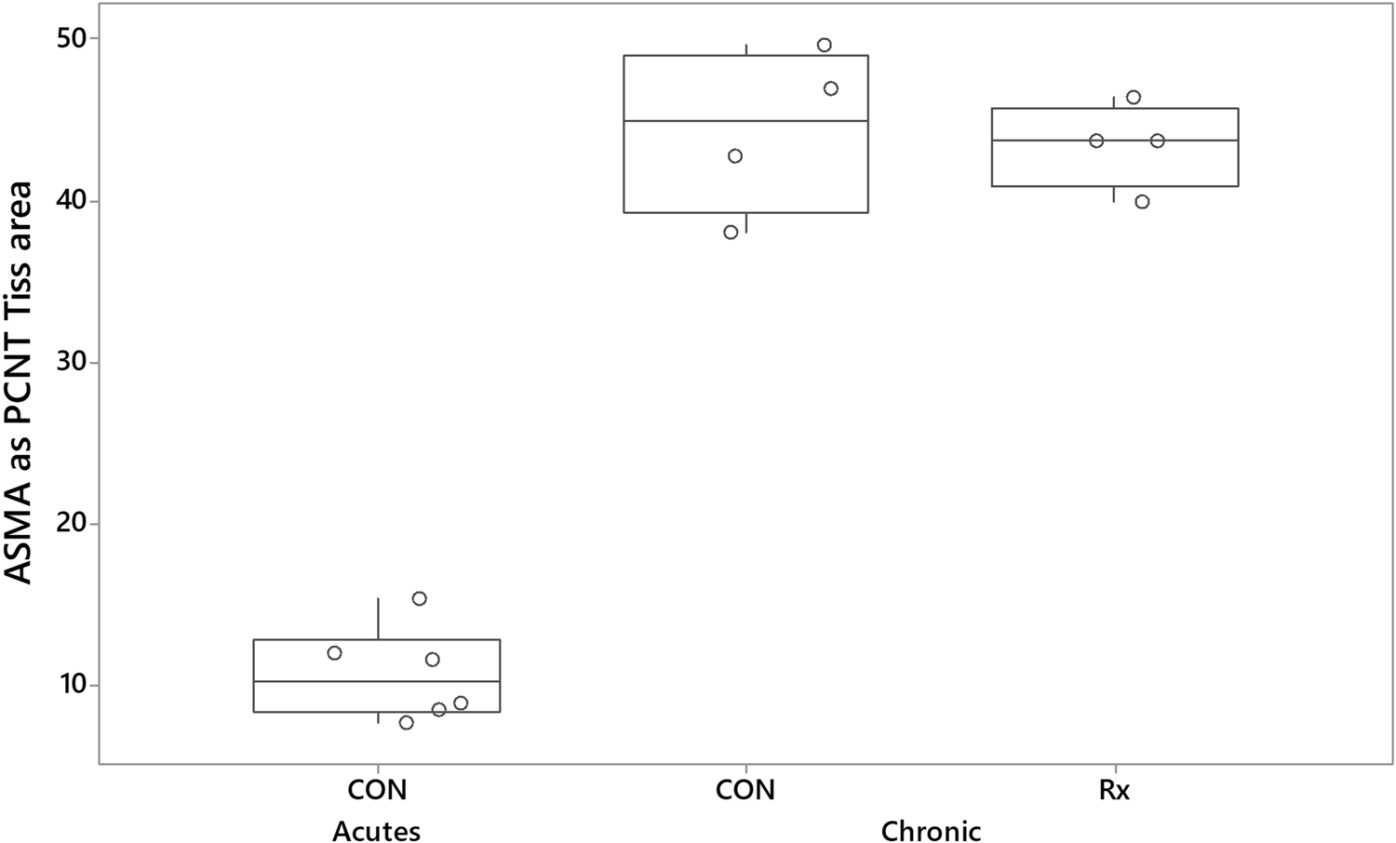
Boxplot illustrating ASMA stain expressed as a percentage of tissue area. The derivation of alpha-smooth muscle actin (ASMA) measurements was as described for Fig. 3. ASMA expression was increased in both radio-exposed (Rx) and contralateral control (CON) lung of sheep undergoing a chronic radiation response, relative to levels seen in the control lungs of sheep undergoing an acute radiation response (CON, Acutes).

Alpha-smooth muscle actin (ASMA) expression was increased equivalently (~ fourfold) in both the chronically responding radio-exposed and contralateral control lungs relative to levels in acutely responding control lungs (Fig. 7). One-way ANOVA indicated that ASMA expression was significantly (P < 0.001) increased in both radio-exposed and contralateral control lungs of sheep responding chronically to radiation relative to levels found in control lungs of sheep responding acutely to radiation.

**Figure 8:**
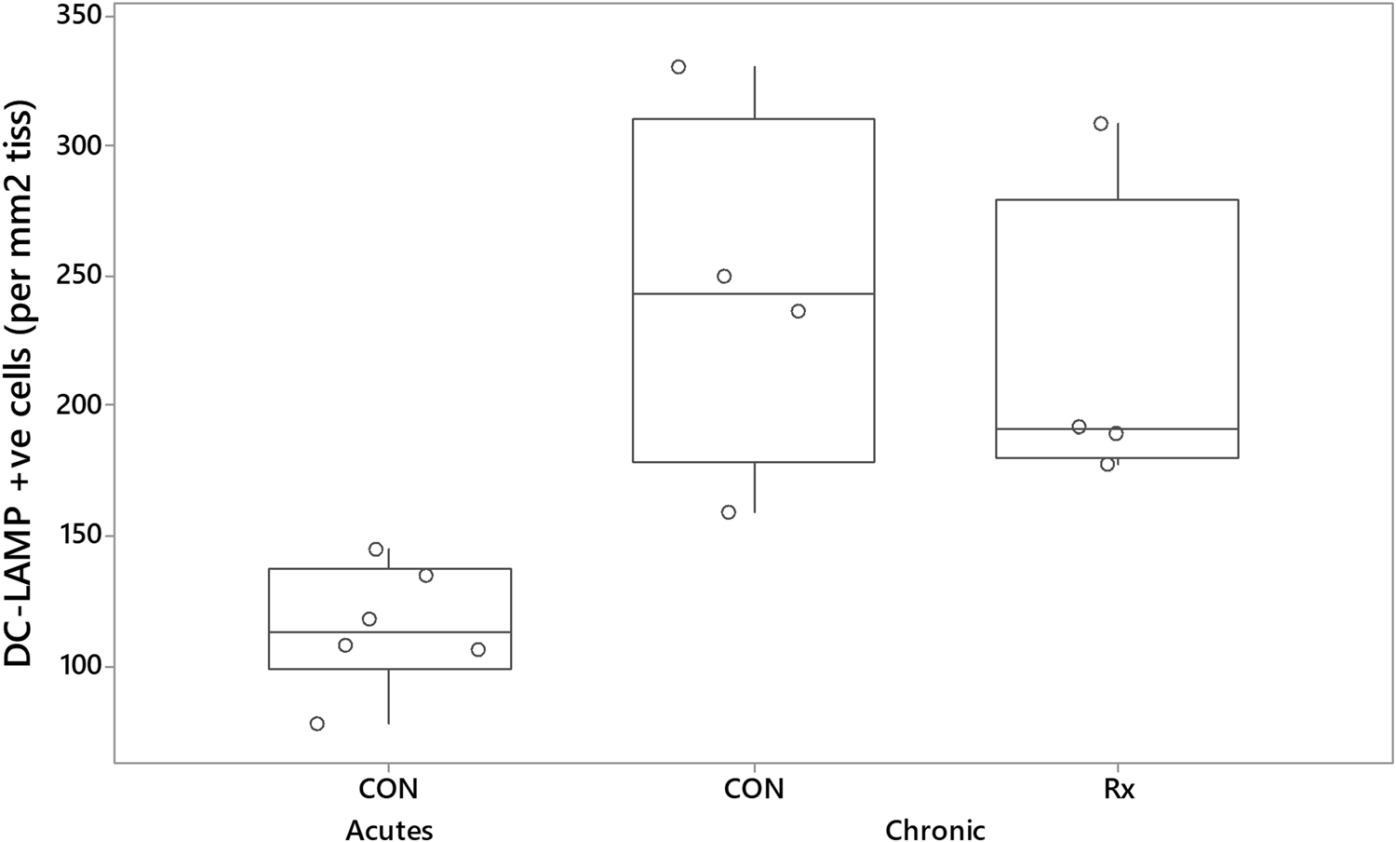
Boxplot illustrating numbers of DC-LAMP positive cells expressed per mm^2^ tissue. The derivation of mean numbers of DC-LAMP positive cells was as described for Fig. 3. The data illustrates that, relative to levels seen in the control lungs of sheep undergoing an acute radiation response (CON, Acutes), there was increased numbers of DC-LAMP positive cells throughout the lungs of sheep undergoing a chronic radiation response.

The number of DC-LAMP expressing cells per unit tissue area approximately doubled in both the radio-exposed and contralateral control lungs relative to numbers in the acutely responding contralateral control lung (Fig. 8). One-way ANOVA indicated that counts of DC-LAMP expressing cells increased significantly (P = 0.005) in both radio-exposed and contralateral control lungs of sheep responding chronically to radiation relative to levels found in control lungs of sheep responding acutely to radiation.

## Discussion

We demonstrate that a targeted, fractionated radiation prescription to a small delineated PTV within the left caudal lung (11% of total lung volume) imposed long-standing impacts on lung structure with increased collagen deposition and reduced vascularity in irradiated lung. Importantly, significant pathologic changes were also noted in un-irradiated lung; namely there was evidence of increased capillary vascularity and increased expression of ASMA, Ki67, and DC-LAMP.

We found an initial increase, followed by a decrease in radio-density in the radio-exposed lung relative to the contralateral control lung. These observations bear comparison with those of Kahán et al., in which breast cancer patients had significantly lower lung density 1 year after radiotherapy compared to density at 3 months^47^. The reasons for the reduced radio-density in the present model are open to conjecture, but intuitive reasoning would tend to associate it with the concomitant variation in vascular filling. With imaging studies confirming that radiation exposure compromises lung vascular perfusion for at least a year post radiotherapy^48^ we suggest that the reduced radio-density in the present model may reflect reduced lung perfusion.

Pulmonary texture analysis offers the facility to objectively quantify change that may not be visible to the radiologist. The application of such texture-based radiomic features to differentiate and stage human lung disease including malignancies and interstitial disorders has significant potential^49,50^. Texture features of the pulmonary parenchyma from radio-exposed and contralateral lung slices were evaluated using the GLCM method and homogeneity found to be significantly lower in radio-exposed lung (relative to contralateral control) at d21, and higher at d171–227. Whilst it is tempting to speculate that the latter increase in homogeneity reflects the reduction in vascularity and perfusion that featured in the necropsy samples, it was beyond the scope of the present study to accommodate the methodology to address this hypothesis. However, the benefits of studies designed to tie together longitudinal in vivo measurements of pulmonary perfusion with concurrent evaluation of texture-based radiomic features are obvious. Indeed such studies would also benefit from the option of exploring tissue level correlates using microCT, histological and indeed spatial molecular analysis at necropsy.

We found an increase in the expression of the endothelial cell marker CD31 in both radio-exposed and contralateral lung relative to levels seen in the contralateral lung during the acute response. We also found a negative correlation between collagen deposition and CD31 expression. Such findings compare favourably with the observations of Ebina et al. (2004), in that biopsies from patients with usual interstitial pneumonia had significantly increased capillary vascular density in areas with low grades of fibrosis relative to control lungs, whereas vascular density gradually decreased as the degree of fibrosis increased, with no vessels observed inside fibroblastic foci^31^. Further, in systemic sclerosis related pulmonary fibrosis, the histopathologic feature of the pulmonary microcirculation best associated with pulmonary hypertension was capillary proliferation in areas without interstitial fibrosis^32^. These, and other reports highlight the capacity for microvascular remodelling in progressive-fibrosing interstitial lung diseases (ILDs)—including in non-fibrotic areas of the lung^33,34^.

The concept that vascular remodelling plays a causative role in driving fibrosis, has been posited by Puxeddu et al.^51^, who highlighted studies demonstrating that aspects of pulmonary venous occlusive disease (PVOD) and capillary haemangiomatosis are seen in areas unaffected by fibrosis in IPF^31,33,34^. In addition, such conditions feature the accumulation of haemosiderin-laden alveolar macrophages^33,34,52^, suggesting compromise of the alveolar-capillary barrier. Our histopathological assessment, in confirming the presence of pigment laden intra-alveolar histiocytes in several sheep, supports these observations.

Recent data identifying specific variants of neoangiogenesis in usual interstitial pneumonia (UIP), non-specific interstitial pneumonia (NSIP) and alveolar fibroelastosis (AFE) demonstrate distinct and specific differences in the morphology of vascular remodelling and associated gene expression profiles between these conditions^53^. Whilst this data related to diseased areas of the lung, the exciting possibility is that nuances in the microvascular response might also pre-empt these specific pathologies and provide avenues towards understanding the interplay between putative insults and the role of host genetics in mediating the responses.

Our observation that microvasculature out with the radiation field failed to perfuse with PBS suggests that low resistance perfusion pathways were to be found in the radiation field. Such pathways, representing anastomoses between the systemic (bronchial) and pulmonary circulations, can arise at precapillary, capillary, or post-capillary levels and serve to shunt pulmonary arterial blood around non-patent capillary beds.

In considering the mechanisms underlying organ-level responses, the acute RILI-evoked mobilisation and spread, in the blood and lymphatic circulation, of cells and extracellular vesicles and mediators (including hypoxia-induced factors, miRNAs, pro-inflammatory cytokines, and damage-associated molecular patterns (DAMPs)), all carry the potential for systemic impact. In addition, the local interaction of bioactives such as neuropeptides and neurotransmitters with neuronal, immune and tissue-resident cell populations can potentially mediate effects through local neural networks as well as with the spinal cord and brain, with potentially widespread effects^54^. Acknowledging that local tissue injury can signal both organ-level and systemic responses, the conundrum remains that such responses in concert with local events not only fail to adequately repair radiation-induced damage, but lead to chronic and functionally detrimental changes to tissue architecture. Intuitively, consideration turns to local mechanisms that could account for this observation.

Relative to levels measured in the control lungs of sheep during the acute phase of the radiation response, we observed approximately four-fold increased levels of expression of alpha smooth muscle actin (ASMA) and approximately two-fold increased numbers of cells expressing dendritic cell lysosomal associated membrane protein (DC-LAMP) throughout the lung. ASMA is the isoform of actin present within vascular smooth muscle cells and in myofibroblasts, a morphologically and metabolically distinct differentiate of fibroblasts that, when activated, synthesise extracellular matrix proteins and collagen^55^. Whilst the expression of ASMA is often taken as evidence of activation and fibrogenesis in the lung, recent studies have challenged this and determined that ASMA is an inconsistent marker of fibrogenesis^56^. Indeed, a subpopulation of collagen producing cells, characterized by the expression of Cthrc1 (collagen triple helix repeat containing 1) is responsible for expressing the highest levels of collagen in fibrotic lungs, whereas cells expressing the highest levels of ASMA expression (smooth muscle cells and pericytes), display lower levels of collagen expression^57^. Given the similar lack of concordance between ASMA expression and collagen production seen in this study, the question remains as to what cell types are responsible for the increased expression in this model. The increased expression of the AEC2 marker DC-LAMP may also be linked to the increased vascularity. Such cells are considered to be the main source of vascular endothelial growth factor (VEGF) in the lung parenchyma^58^. VEGF is a potent regulator of vascular permeability, a key player in angiogenesis, and a survival factor for endothelial cells^59^. Notably Ebina et al. found augmented expression of VEGF in capillary endothelial cells and alveolar Type II epithelial cells in the highly vascularized alveolar septa of minimally fibrotic areas of IPF lungs^31^. Increased cell proliferation was seen throughout the lungs undergoing a chronic radiation response. In the contralateral lung, such response might also relate to the increased vascularity, whereas in the radio-exposed lung, increased cell proliferation likely reflects adaptation to the pathology.

These important local and organ-wide changes were demonstrated following treatment with a moderate hypofractionated, conformal radiation protocol. The changes we identified may not be identical to pathologic changes following other radiotherapy approaches such as intensity modulated radiation therapy (IMRT), where more normal tissue generally receives low dose radiation^60,61^, thus complicating the identification of pathways to non-target lung injury. This protocol was initially selected due to the desire to utilize a clinically translatable protocol, while balancing a need to avoid adverse events that negatively affected the quality of life of the animals following radiotherapy and repeated anaesthesia.

We have therefore shown for the first time that the chronic lung response to radiation in sheep involves responses in both radio-exposed and non-exposed lung. The generalised increase in expression of endothelial, AECII, myofibroblast, and proliferating cell markers indicate that certain aspects of the chronic response to local lung radiation are levelled organ-wide. However, other changes, namely increased deposition of collagen and evidence of reduced microvascular perfusion, were specific to the radiation field and equally, an increase in vascularity was apparent only in non-radio-exposed lung. Our conclusion is that pathological changes in the radiation field likely reflect the interaction of both organ-wide responses, and those originating and enacted at the local level. Concepts emerging from these observations can influence strategies aimed at preventing lung fibrosis. For instance, knowledge that microvascular perturbation is negatively associated with collagen deposition suggests value in strategies aimed at characterising and restoring perfusion deficits post radiation exposure. These efforts to discern temporal and consequential changes in a relevant sheep model provide momentum towards the development of mitigation strategies for the avoidance of RILI while maintaining or improving tumour control with radiation therapy.

## Data Availability

The datasets generated and analyzed during the current study are available from the corresponding author on reasonable request.
